# Young Male With Seizure Disorder and Intracranial Calcification: A Case of Fahr’s Syndrome

**DOI:** 10.7759/cureus.22189

**Published:** 2022-02-14

**Authors:** Jithesh G, Rifika Bansal, Mohammed Ajmal, Paras Gupta, Monika Pathania

**Affiliations:** 1 Internal Medicine, All India Institute of Medical Sciences, Rishikesh, IND; 2 General Medicine, All India Institute of Medical Sciences, Rishikesh, IND

**Keywords:** hypoparathyroidism, seizure, intracranial calcification, hypocalcemia, fahr’s syndrome

## Abstract

Fahr’s syndrome is a neurodegenerative disorder characterized by abnormal deposition of calcium in the brain, especially in basal ganglia. The term Fahr’s disease is used when primary familial brain calcification is present, and the term Fahr’s syndrome is used for secondary causes. Our patient is a 35-year-old male who presented to our hospital with complaints of two episodes of generalized tonic-clonic seizures. He had a history of recurrent episodes of seizures since the age of 15 and they all were generalized tonic-clonic seizures. He did not have a family his­tory of epilepsy. Lab investigations showed a normal hemogram, and liver and renal function were within normal limits. Serum electrolyte levels showed hypocalcemia, but other electrolyte levels were normal. He had low parathyroid hormone levels and normal levels of vitamin D. Brain imaging studies with non-contrast CT and a contrast-enhanced MRI showed bilaterally symmetrical dense calcifications. The etiology in our patient was the primary hypoparathyroidism and was treated accordingly. He reported symptomatic improvement with treatment and had no episodes of seizures after the commencement of the treatment. So, in cases of Fahr’s syndrome, treatable etiologies must be ruled out as they can delay the progression of the disease.

## Introduction

Fahr’s syndrome is a neurodegenerative disorder that is characterized by abnormal deposition of calcium in the brain, especially in basal ganglia. The term Fahr’s disease is used when primary familial brain calcification is present, and the term Fahr's syndrome is used for secondary causes. The prevalence of Fahr’s syndrome is one in 1,000,000 [1]. Several conditions have been noted among etiologies, most common being endocrine disorders, dermatological abnormalities, infectious diseases, and mitochondrial myopathies [2,3].

Here we are reporting a case of Fahr’s syn­drome secondary to hypo­parathyroidism in a patient presenting with seizures. Even though the management of this disease is conservative, the cases which occur secondary to hypoparathyroidism respond well to the correction of levels of calcium and phosphate. So, in the case of Fahr's syndrome, treatable etiologies must be ruled out as they can delay the progression of the disease.

## Case presentation

**Figure 1:**
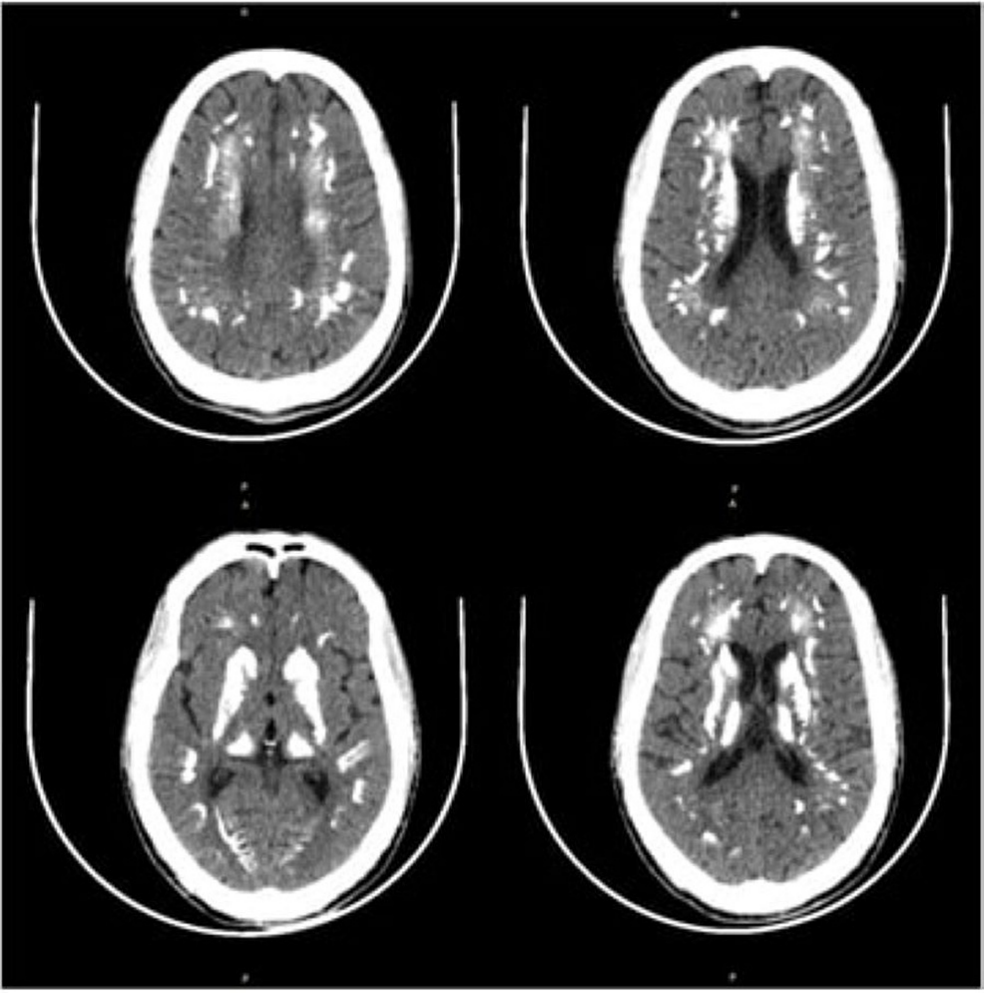
Non-contrast CT of the brain (axial CT images) Bilateral symmetrical, dense, and extensive calcification in subcortical white matter, periventricular gray matter, centrum semiovale, caudate nucleus, basal ganglia, internal capsule and thalamus.

**Figure 2:**
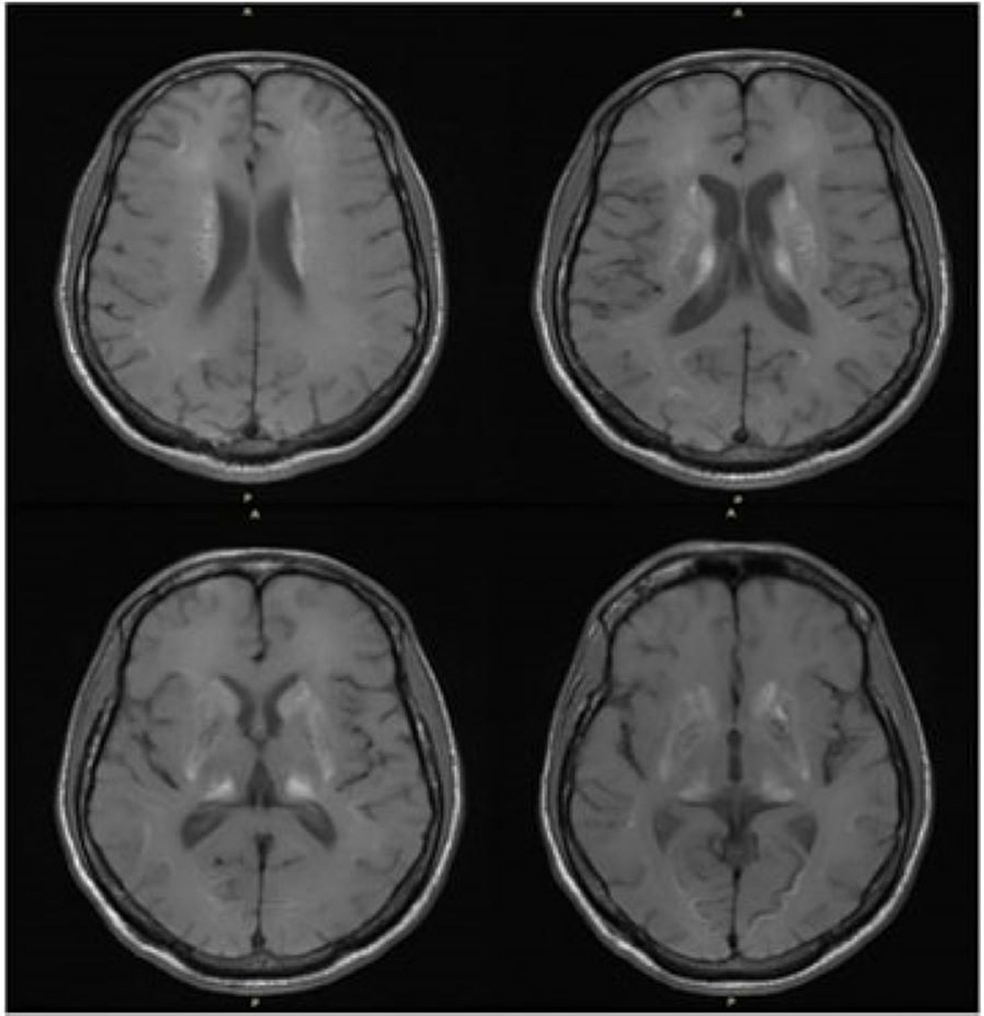
Axial MRI brain T1 hyperintensities bilateral caudate nuclei, thalami, subthalamic nuclei, and substantia nigra.

A 35-year-old male, resident of the Himalayan region, Northern India, farmer by occupation, presented to our hospital with complaints of two episodes of the generalized tonic-clonic seizure (GTCS). As per the history narrated by his brother, the movements appeared like tonic-clonic movements of limbs. The episode lasted for approximately 15 minutes following which he remained in a state of altered consciousness. He regained his consciousness after 1 hour from the episode. He did not have any history suggestive of head trauma, fever, or symptoms suggestive of central ner­vous system infection, focal neurological deficit. There was no history of hypertension, diabetes, thyroid disease, or autoimmune disease. He gave a history of recurrent episodes of seizures since the age of 15 years which were all GTCSs, 1-2 episodes in a year, associated with postictal confusion and urinary incontinence. He was never on proper antiepileptic treatment and he was intermittently following an alternative system of medicine from local practitioners for the same. Since seizure used to be self-limiting, due to lack of resources in this remote hilly area, poor socioeconomic status, and local belief of seizure being confused with a curse of god, he never went to hospital for evaluation of his symptoms or disease. This time he was taken to hospital as he was not regaining consciousness like he used to, in his earlier days. The last seizure episode was reported 6 months back. He did not have any family his­tory suggestive of epilepsy. On examination, he had normal vitals and normal temperature. Neurologic evaluation at admission showed the patient in a postictal state, with no cranial nerve impairment and focal neurologic deficit. Laboratory investigations showed a normal hemogram, and liver and renal functions were within normal limits. His serum electrolyte panel showed hypocalcemia with the rest of the electrolytes within normal limits. Further workup revealed low normal serum levels of vitamin D, but low levels of parathyroid hormone (PTH) (Table 1). Our patient had a seizure disorder with hypocalcemia due to hypoparathyroidism. Brain imaging was done to look for any other cause and it showed extensive bilateral cerebral calcification (Figures 1-2). The first differential was kept of Fahr’s syndrome. The usual age of onset of the disease is the fourth decade in the case of Fahr’s syndrome and here the symptoms started from the second decade. The possibility of Fahr’s disease was ruled out as there is no family history of similar illness and an obvious secondary cause was present. No other causes for Fahr’s syndrome other than primary hypoparathyroidism were present. Primary hypoparathy­roidism was diagnosed based on low calcium, high phosphate, low PTH, and normal vitamin D levels. The only manifestation in our patient was a seizure. There were no features suggestive of perioral numbness, paresthesia, muscle cramps, carpopedal spasm, laryngospasm, and prolonged QT interval. There was no evidence of secondary causes of hypoparathyroidism such as the history of congenital anomalies, autoim­mune disease, neck irradiation, thyroid surgery, or any features of hypomagnesemia. The possibility for intracerebral calcification secondary to TORCH (Toxoplasmosis, Other (syphilis, varicella-zoster, parvovirus B19), Rubella, Cytomegalovirus (CMV), and Herpes) infections was ruled out with the age of presentation, uneventful antenatal history, and absence of a history of any developmental delay. He did not have any dermatological manifestations, and lactate levels were normal in his cerebrospinal fluid, ruling out the possibility of Mitochondrial Encephalomyopathy Lactic acidosis Stroke-like episodes (MELAS), and other secondary causes of Fahr’s syndrome. He was administered a loading dose of levetiracetam (1 g) intravenously and maintained on oral levetiracetam (500 mg) twice daily. Because of hypocalcemia and seizures correction of hypocalcemia was performed. He was started on calcium correction measures initially administered as an intravenous infusion and later changed to oral medications (elemental calcium 500 mg twice a day) with oral calcitriol (0.5 µg once daily). He was monitored for symptom recurrence and was discharged on oral levetiracetam, oral calcium, and oral calcitriol supplements once he improved symptomatically. He is on regular follow up after discharge on an outpatient basis and reported no recurrence of symptoms and experiences a significant improvement in overall quality of life.

**Table 1:** Laboratory data PTH: parathyroid hormone

Laboratory Data	Admission	Discharge	Reference Range
Serum calcium	1.5 mmol/L	2.1 mmol/L	2–2.5 mmol/L
Corrected serum calcium	1.7 mmol/L	2.2 mmol/L	
Vitamin D	20 ng/mL	27 ng/mL	20–40 ng/mL
PTH	4.5 pg/mL	9 pg/mL	10–55 pg/mL
Phosphate	1.6 mmol/L	1.3 mmol/L	0.8–1.5 mmol/L

## Discussion

Fahr’s syndrome is also known as idiopathic basal ganglia calcification, bilateral striopallidodentate calcinosis (BSPDC), and calcinosis nucleorum. It was first reported in 1930 by Karl Theodor Fahr [4]. It is a neurodegenerative disorder that is characterized by abnormal deposition of calcium in the brain, especially in basal ganglia. The term Fahr’s disease is used when primary familial brain calcification is present, and the term Fahr’s syndrome is used when there are obvious secondary causes for brain calcification. The most common secondary cause of Fahr’s syndrome is hypoparathyroidism, pseudohypoparathyroidism, hyperparathyroidism, hypervitaminosis D, tuberculosis, cytomegalovirus infection, toxoplasmosis, astrocytoma, and MELAS [2,3]. The mean age of symptom onset is 40 years with men presenting with double the prevalence rate as women [5]. Our patient was having symptoms from the age of 15 years. Calcifications commonly occur in basal ganglia, thalamus, dentate nucleus, cerebral cortex, cerebellum subcortical white matter, and hippocampus [6,7] as was in our case. Deposits are composed of mineral compounds like calcium phosphate and carbonate, glyconate, mucopolysaccharide, and metals including iron, copper, magnesium, zinc, aluminum, silver, and cobalt may also be found [8,9]. The most common neurologic symptoms include seizure, movement disorder such as parkinsonism, chorea, tremor athetosis, gait impairment, speech impairment psychiatric manifestation in the form of depression, and psychosis [1]. The treatment of the disease is usually symptomatic. But it has been found that seizure and movement disorder in Fahr’s syndrome due to hypoparathyroidism usually responds well with the correction of phosphate and calcium levels [10,11].

## Conclusions

General practitioners in hilly resource-poor settings should always consider uncommon etiologies of seizure as well in order to prevent complications due to the same. Detailed counseling regarding the nature of the disease ensures compliance with antiepileptic drugs. Early diagnosis may lead to correction of electrolyte disturbance which prevents inadvertent use of antiepileptic drugs and their adverse events in the future. Public awareness regarding seizures and the importance of treatment needs to be done in resource-poor settings.
